# “Too much, too soon?” Early professional match exposure and career sustainability: a 10-year multileague cohort of professional female soccer players

**DOI:** 10.1186/s13102-026-01652-6

**Published:** 2026-04-01

**Authors:** Ibrahim Ebeid

**Affiliations:** Faculty of Medicine, Menoufia University, Shebin El-Kom, Menoufia Egypt

**Keywords:** Women’s soccer, Early professional exposure, Career sustainability, Athlete development, Survival analysis

## Abstract

**Background:**

Debate persists about whether early professional competition compromises long-term career sustainability in youth athletes; evidence in women’s professional soccer is limited.

**Hypothesis:**

We hypothesized that greater cumulative professional match exposure before age 18 would be associated with increased risk of sustained attrition between ages 18 and 23.

**Study design:**

Retrospective cohort study.

**Methods:**

A retrospective cohort study was conducted using a large multinational cohort of professional female soccer players competing in top-tier leagues in England, France, Germany, and Italy between 2010 and 2020. Players who made their professional debut before age 18 were included. Pre-18 exposure was categorized by cumulative club minutes: ≤250, 251–750, 751–1,750, or > 1,750 min. The primary outcome was sustained attrition, defined as a **≥** 12-month absence from professional competition between ages 18 and 23. Analyses included Kaplan–Meier methods, Cox proportional hazards models, and multivariable regression adjusting for debut age, position, and height.

**Results:**

Of 1,148 players, 406 (35.4%) experienced sustained attrition between ages 18 and 23. Higher pre-18 exposure was associated with a lower likelihood of sustained attrition: adjusted hazard ratios were 0.60 (95% CI, 0.47–0.75) for 251–750 min, 0.37 (95% CI, 0.28–0.50) for 751–1,750 min, and 0.26 (95% CI, 0.17–0.38) for > 1,750 min (*P* < 0.05). Greater pre-18 exposure was also independently associated with higher mean annual playing time and greater match participation between ages 18 and 23.

**Conclusion:**

Among professional female soccer players, higher levels of professional match exposure before age of 18 were associated with improved career sustainability. These findings suggest that players capable of accumulating high match volumes in early professional environments are more likely to maintain long-term careers. Rather than viewing early professional competition as an inherent risk, these results support carefully supervised early integration as a means to identify and develop robust athletic trajectories.

**Level of evidence:**

Level 3.

## Introduction

Participation in organized elite youth soccer has increased rapidly, and many national associations now operate structured talent identification and development programs aimed at accelerating the transition from promising adolescent athlete to senior professional. These programs commonly employ standardized motor testing, coach ratings, and monitored competitive exposure to inform selection and individualized development plans [1, 2].

Female players have been historically under-represented in soccer-specific talent research despite the rapid professionalization of the women’s game and demonstrable sex-specific patterns in injury, development, and competitive exposure. Prospective, longitudinal studies examining multidimensional predictors (objective motor tests, coach-rated technical/tactical skills, and psychosocial factors) and their predictive validity for selection at successive levels and adult professional attainment are scarce [2–4].

A parallel concern among clinicians and sport scientists is the potential harm of excessive early exposure and early single-sport specialization. Systematic reviews and consensus statements associate early specialization and very high training or competition loads in childhood or early adolescence with increased risk of overuse injury, burnout, and premature dropout, while recognizing that the relationship between early exposure and long-term elite attainment may differ by sport, sex, and developmental context [5–8]. Consequently, practitioners face a practical tension: early integration into professional match environments may accelerate development for some athletes but may also increase risk for others.

Specific to female soccer, longitudinal cohort work has produced mixed findings. Large prospective studies from Germany indicate that single motor measures (e.g., sprinting) can show prognostic relevance, yet motor development trajectories across early adolescence (U12–U15) do not always sufficiently discriminate future professional attainment, suggesting that motor tests alone are imperfect predictors of adult success [4]. More recent multidimensional prospective work focusing on U15 girls has demonstrated that combining objective motor tests with coach-rated tactical and psychosocial assessments can increase explained variance for selection outcomes [3].

Beyond talent diagnostics, evolving evidence from athlete monitoring shows that workload, psychological state, and recovery interact dynamically across season and preseason contexts to influence perceived exertion, adaptation, and potentially injury risk. Studies in collegiate and elite female cohorts report that objective external load and morning psychological stress predict session RPE and that training adaptations across brief pre-season blocks are associated with accumulated accelerations/decelerations and training impulse (TRIMP) [9, 10]. These findings indicate that exposure influences short-term adaptation, but its relationship to long-term career sustainability remains unclear, particularly in women’s professional soccer.

Taken together, the literature highlights well-established concerns regarding early specialization and excessive youth training load, as well as methodological limitations in much of the talent identification research. However, much of this work focuses on academy participation, training volume, or specialization pathways rather than actual professional competitive exposure during late adolescence, which represents a distinct developmental transition. Professional match participation reflects not only training load but also selection processes, competitive readiness, and organizational investment in player development. Prospective studies that integrate these factors and examine the association between pre-18 professional match exposure and post-18 career sustainability in large, multinational female cohorts remain scarce. To address these gaps, we conducted a retrospective cohort analysis of elite female players who debuted professionally before age 18, quantifying cumulative pre-18 professional minutes, multidimensional early performance indicators, and subsequent career trajectories through age 23.

## Methods

### Study design and data sources

**Fig. 1:**
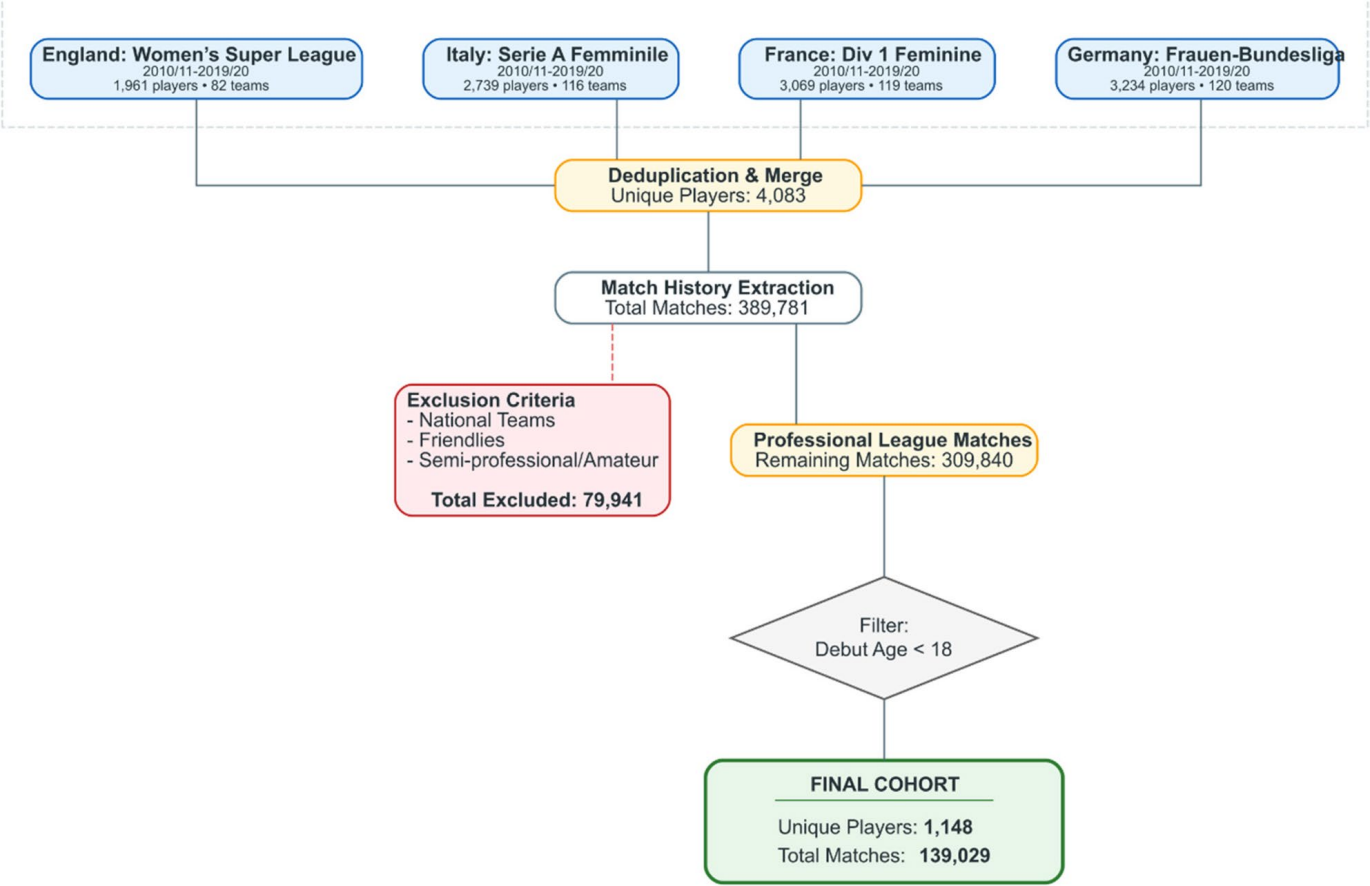
Flowchart of the cohort selection of female professional soccer players

We conducted a retrospective cohort study of professional female soccer players, with data collected from Soccerdonna (https://www.soccerdonna.de/). Soccerdonna is a specialized, publicly available online database that provides comprehensive historical and statistical records for women’s association soccer globally. While Soccerdonna provides comprehensive match participation and transfer records, it is not designed as a clinical or athlete-monitoring database and does not include individual-level medical, biological maturation, training load, psychosocial, or contractual variables. The study population was drawn from the top-tier professional leagues in England (Women’s Super League), France (Division 1 Féminine), Germany (Frauen-Bundesliga), and Italy (Serie A Femminile). Data were collected for all players who participated in these leagues between the 2010–11 and 2019–20 seasons. For the English Women’s Super League, the 2016–17 season was excluded as no full league season occurred during the transition to a winter calendar. Player and match-level data were assembled into a longitudinal dataset containing demographic characteristics, complete competitive match histories, and transfer records (Fig. 1).

### Inclusion and exclusion criteria

The analytic cohort was restricted to players who made their first senior professional appearance before the age of 18 years. From 1,148 eligible players, we extracted granular match-day data, including minutes played per appearance. To ensure the analysis focused strictly on professional club trajectories, we applied the following exclusions: (1) All international matches (Senior, U21, U19, and U17). (2) Matches played in semi-professional or amateur tiers. (3) All friendly matches.

### Study period and follow-up

For each player, follow-up began on the date of their 18th birthday and continued until the earliest of (1) their 23rd birthday or (2) the end of available match data. Only competitive professional club matches occurring within this post-18 follow-up window were considered for outcome assessment. The primary exposure was cumulative professional match participation before the 18th birthday, quantified as total competitive minutes played. Players were categorized into four groups based on pre-18 min: ≤250 min, 251–750 min, 751–1,750 min, and > 1,750 min, representing progressively higher levels of early professional match exposure based on the cohort distribution.

### Outcomes

Primary Outcome (Sustained Attrition): Defined as a continuous absence from competitive professional matches lasting at least 12 months during the post-18 follow-up period, without a subsequent return to play before the end of follow-up. The ≥ 12-month threshold was chosen pragmatically to indicate a prolonged interruption that exceeds a single competitive season across the included top-tier leagues, and therefore to reflect extended discontinuity in professional club participation rather than a typical short-term injury absence or a seasonal gap. Secondary Outcomes: Measures of post-18 career participation, including mean annual competitive minutes, total match count, longest interruption in participation, and number of club transfers.

### Statistical analysis

All statistical analyses were performed using Python (version 3.10), with the significance level set at < 0.05 for all two-sided tests. Baseline characteristics and career metrics were summarized for the total cohort and stratified by pre-18 exposure group, using means, standard deviations (SD), medians, and inter-quartile ranges (IQR) for continuous variables and counts and percentages for categorical data. Group differences were evaluated using parametric or non-parametric tests as appropriate for the data distribution. We analyzed the time to sustained attrition using Kaplan–Meier methods, with survival curves compared via the log-rank test. Multivariable Cox proportional hazards regression was employed to estimate hazard ratios (HRs) and 95% confidence intervals (CIs) for the association between pre-18 match exposure and attrition, adjusting for age at debut and playing position. To further evaluate the risk of attrition, multivariable logistic regression models provided odds ratios (ORs) for the binary outcome. For post-18 participation metrics, we utilized generalized linear models with a Gamma distribution and log link to account for the right-skewed nature of annual minutes, while negative binomial regression was used for match counts to address overdispersion. All primary models were adjusted for age at debut and playing position (goalkeeper, defender, midfielder, or striker), with sensitivity analyses incorporating height where data were available. Model assumptions and fit were verified using standard diagnostic procedures, including assessment of proportional hazards and residual analysis.

### Ethical considerations

As this study utilized publicly available data without personal identifiers, formal ethical approval was not required under current research guidelines.

## Results

### Study population and baseline characteristics

The study cohort comprised 1,148 female professional soccer players who debuted before age 18 and were followed through age 23 where available. Pre-18 professional minutes were distributed as follows: ≤250 min (*n* = 427), 251–750 min (*n* = 318), 751-1,750 min (*n* = 244), and > 1,750 min (*n* = 159). Mean age at professional debut and height differed significantly across exposure groups (*p* < 0.001, *p* = 0.003). (Table 1.)

**Table 1:** Baseline characteristics by pre-18 professional exposure group (minutes)

Characteristic	Overall (*N* = 1148) mean ± SD; median [IQR] or n (%)	<=250 (*n* = 427) mean ± SD; median [IQR] or n (%)	251–750 (*n* = 318) mean ± SD; median [IQR] or n (%)	751–1750 (*n* = 244) mean ± SD; median [IQR] or n (%)	> 1750 (*n* = 159) mean ± SD; median [IQR] or n (%)	*p* value
Age at debut (years)	16.9 ± 0.7; 16.9 [16.5–17.4]	17.1 ± 0.7; 17.3 [16.7–17.6]	17.0 ± 0.6; 17.1 [16.6–17.5]	16.7 ± 0.6; 16.8 [16.5–17.1]	16.2 ± 0.6; 16.3 [15.9–16.6]	< 0.001
Height (cm)	168.7 ± 5.8; 168.0 [165.0–173.0]	167.8 ± 5.9; 167.0 [164.0–171.0]	168.4 ± 5.7; 168.0 [165.0–172.0]	169.2 ± 5.7; 169.0 [165.0–173.0]	169.8 ± 5.8; 170.0 [166.0–174.0]	0.003
Position						
Goalkeeper	103 (9.0%)	33 (7.7%)	41 (12.9%)	18 (7.4%)	11 (6.9%)	0.115
Defence	385 (33.5%)	134 (31.4%)	108 (34.0%)	78 (32.0%)	65 (40.9%)	
Midfield	417 (36.3%)	166 (38.9%)	107 (33.7%)	92 (37.7%)	52 (32.7%)	
Striker	243 (21.2%)	94 (22.0%)	62 (19.5%)	56 (23.0%)	31 (19.5%)	
Has full follow-up to 23						
No	18 (1.6%)	9 (2.1%)	7 (2.2%)	2 (0.8%)	0 (0.0%)	0.168
Yes	1130 (98.4%)	418 (97.9%)	311 (97.8%)	242 (99.2%)	159 (100.0%)	

*Abbreviations*: *SD* Standard Deviation, *IQR* Inter-quartile Range

### Sustained attrition between ages 18 and 23

**Fig. 2:**
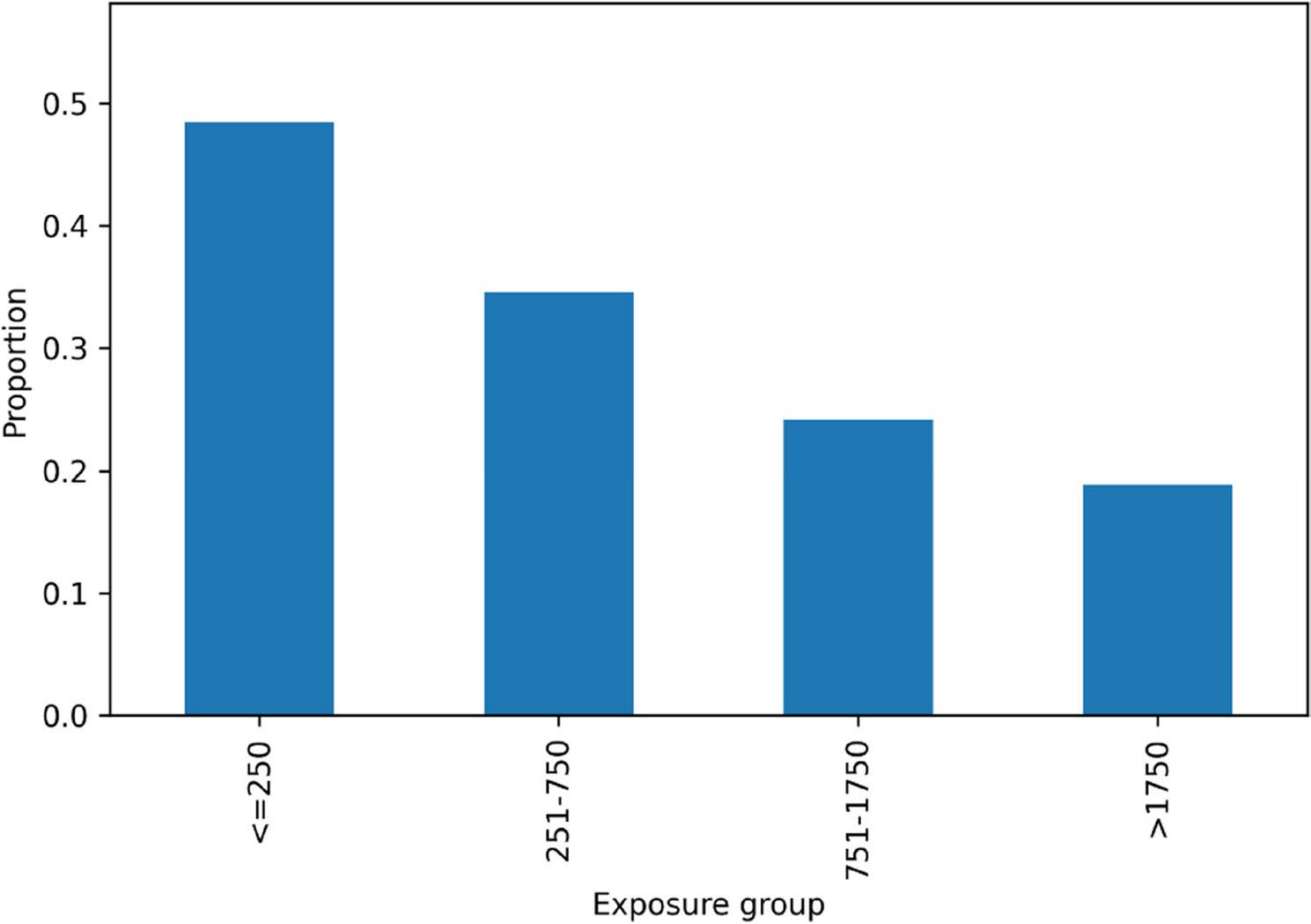
Proportion of sustained attrition among professional female soccer players by pre-18 exposure group

Overall, sustained attrition occurred in 406 of 1,148 players (35.37%). Attrition proportions decreased across higher exposure categories (Fig. 2). Post-18 to 23 availability metrics, including longest gap in competitive match participation and the proportion experiencing a ≥ 12-month gap with return, are shown in Table 2.

**Table 2:** Career outcomes and participation metrics (ages 18–23) by pre-18 professional match exposure group

Outcome	Overall (*N* = 1148) mean ± SD; median [IQR] or *n* (%)	≤ 250 (*n* = 427) mean ± SD; median [IQR] or *n* (%)	251–750 (*n* = 318) mean ± SD; median [IQR] or *n* (%)	751–1,750 (*n* = 244) mean ± SD; median [IQR] or *n* (%)	> 1,750 (*n* = 159) mean ± SD; median [IQR] or *n* (%)
Attrition	406 (35.4%)	207 (48.5%)	110 (34.6%)	59 (24.2%)	30 (18.9%)
≥ 12-month gap with return	423 (36.9%)	175 (41.0%)	128 (40.3%)	77 (31. 6%)	43 (27.0%)
Annual minutes	707.8 ± 585.1; 627.2 [165.3–1154.4]	464.2 ± 498.5; 270.0 [9.0–840.7]	656.6 ± 528.6; 553.1 [198.2–1044.7]	924.9 ± 583.7; 963.3 [436.5–1354.8]	1131.1 ± 558.2; 1157.2 [728.3–1560. 9]
Annual matches	9.8 ± 7.6; 9.4 [2.6–16.0]	6.6 ± 6.8; 4.2 [0.2–11.8]	9.2 ± 7.0; 8.4 [3.0–14.8]	12.7 ± 7.3; 13.4 [6.6–19.0]	15.0 ± 6.7; 15.4 [11.2–19.5]
Longest gap between matches (days)	772.8 ± 578.1; 575.5 [277.0–1197.3]	1001.2 ± 607.4; 922.0 [432.5–1686.5]	770.4 ± 546.5; 612.0 [301.0–1090.8]	568.5 ± 484.1; 360.5 [206.3–807.0]	477.5 ± 422.3; 302.0 [173.5–632.0]
Transfers recorded	2.1 ± 1.7; 2.0 [1.0–3.0]	2.1 ± 1.9; 2.0 [1.0–3.0]	2.1 ± 1.7; 2.0 [1.0–3.0]	1.9 ± 1.4; 2.0 [1.0–3.0]	2.0 ± 1.7; 2.00 [1.0–3.0]
≥ 3 club changes	365 [31.8%]	140 (32.8%)	108 (34.0%)	73 (29.9%)	44 (27.7%)

*Abbreviations*: *SD* Standard Deviation, *IQR* Inter-quartile Range

### Time-to-event analyses

**Fig. 3:**
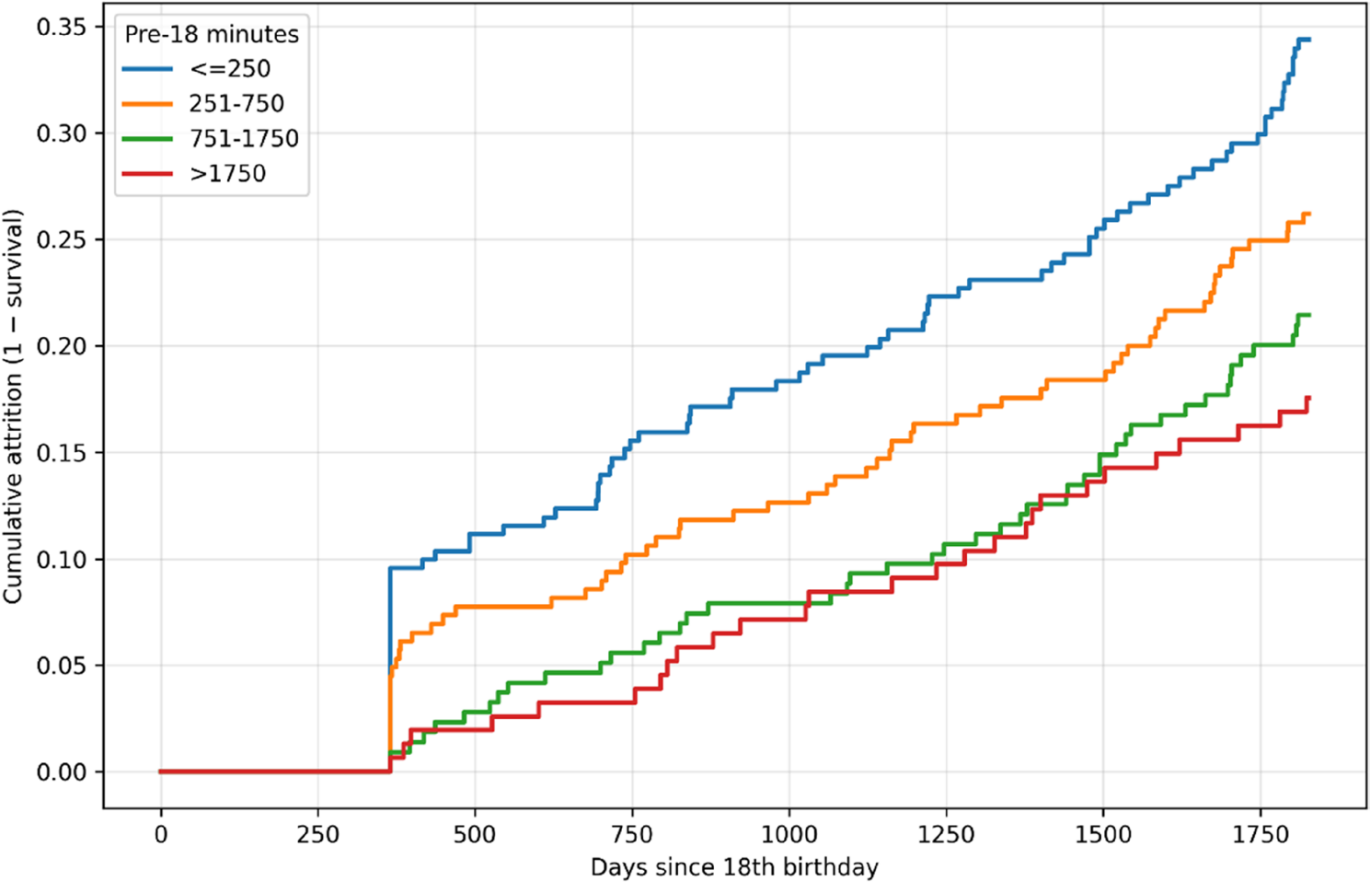
Kaplan-Meier cumulative attrition by pre-18 exposure group

Kaplan-Meier estimates were generated in the subset with complete covariate data for height (*n* = 865; 223 events). Cumulative attrition by the end of follow-up was 34.37% in the ≤ 250-minute group, 26.19% in the 251-750-minute group, 21.42% in the 751-1,750-minute group, and 17.53% in the > 1,750-minute group (Fig. 3). Survival curves differed across exposure groups (log-rank χ² = 18.45, df = 3, *p* < 0.05). In Cox proportional hazards models adjusted for age at debut and position, higher pre-18 exposure was associated with lower attrition hazard. Results were directionally similar in the complete-case sensitivity model additionally adjusted for height (Table 3).

**Table 3:** Kaplan–Meier attrition estimates and Cox proportional hazards models for time to sustained attrition (ages 18–23), stratified by pre-18 exposure group

Exposure group (pre-18 min)	KM baseline *N*	KM events	KM cumulative attrition (%)	Cox Primary HR (95% CI)	*p* value	Cox Sensitivity HR (95% CI)	*p* value
251–750	245	64	26.19	0.60 (0.47–0.75)	< 0.001	0.69 (0.50–0.96)	0.027
751-1,750	215	46	21.42	0.37 (0.28–0.50)	< 0.001	0.56 (0.38–0.81)	0.002
> 1,750	154	27	17.53	0.26 (0.17–0.38)	< 0.001	0.43 (0.27–0.69)	< 0.001

Reference category: ≤250 Exposure Group

*Abbreviations*: *KM*  Kaplan-Meier, *HR*  Hazard Ratio, *CI*  Confidence Interval

### Logistic regression analyses

In multivariable logistic regression adjusted for age at debut and position, the odds of sustained attrition were lower in the 251-750-minute group (OR 0.54, 95% CI 0.40–0.73; *p* < 0.001), the 751-1,750-minute group (OR 0.31, 95% CI 0.22–0.45; *p* < 0.001), and the > 1,750-minute group (OR 0.21, 95% CI 0.13–0.33; *p* < 0.001) compared with the ≤ 250-minute reference. In the complete-case sensitivity model additionally adjusted for height (*n* = 865), exposure estimates were similar (Table 4).

**Table 4:** Adjusted association of pre-18 exposure with sustained attrition (logistic regression). Primary model adjusted for age at debut and position; sensitivity model additionally adjusted for height (complete cases)

Exposure group	Primary model OR (95% CI)*	*p* value	Sensitivity OR (95% CI)†	*p* value†
251–750	0.54 (0.40–0.73)	< 0.001	0.65 (0.43–0.96)	0.030
751–1,750	0.31 (0.22–0.45)	< 0.001	0.52 (0.33–0.80)	0.003
> 1,750	0.21 (0.13–0.33)	< 0.001	0.38 (0.22–0.67)	< 0.001

Reference category: ≤250 Exposure Group

*Abbreviations*: *OR*  Odds Ratio, *CI*  Confidence Interval, *Min* Minutes

^*^Primary model adjusted for age at debut and Position

^†^Sensitivity model additionally adjusted for Height (complete-cases)

### Post-debut playing time and match participation

In complete-case adjusted models including age at debut, position, and height, higher levels of pre-18 professional match exposure were associated with increased mean annual playing time, as well as with greater match participation between the ages of 18 and 23. These associations were consistent across modeling approaches, including Gamma generalized linear models for annual minutes and negative binomial models for match counts (Table 5).

**Table 5:** Adjusted associations of pre-18 exposure with post-18 match participation outcomes (ages 18–23)

Exposure group (pre-18 min)	Mean annual minutes age 18–23 (Gamma ratio, 95% CI)	*p* value	Matches age 18–23 (NegBin IRR, 95% CI)	*p* value
251–750	1.19 (1.06–1.34)	0.003	1.25 (1.05–1.50)	0.014
751-1,750	1.47 (1.30–1.66)	< 0.001	1.55 (1.28–1.88)	< 0.001
> 1,750	1.81 (1.56–2.09)	< 0.001	1.86 (1.48–2.34)	< 0.001

Models adjusted for age at debut, position, and height; analyses restricted to players with complete height data. Ratios/IRRs are relative to the < = 250-minute reference group

Reference category: ≤250 Exposure Group

*Abbreviations*: *CI*  Confidence Interval, *NegBin IRR*  Negative Binomial Incidence Rate Ratio

## Discussion

This cohort elucidated a robust association between early professional match exposure and long-term career sustainability in professional female soccer players. Among 1,148 players, higher cumulative professional minutes prior to age 18 were associated with a progressively lower likelihood of sustained attrition during the critical transition period between ages 18 and 23. Specifically, players accumulating more than 1,750 min of professional match exposure before age 18 exhibited the lowest hazard of sustained attrition between ages 18 and 23 (HR = 0.26; 95% CI 0.17–0.38), representing an approximately 74% lower attrition hazard compared to the lowest exposure group. While much of the existing clinical literature emphasizes the potential risks of early specialization and high match loads, our findings demonstrate a divergent pattern in this elite cohort. This suggests that within women’s professional soccer, early integration may not be a primary driver of attrition but rather an indicator of a player’s capacity to navigate the youth-to-senior transition. This apparent paradox is likely explained by the selection and survivor biases inherent to elite professional environments; thus, these findings do not establish causation and instead suggest that higher match loads in late adolescence are not uniformly associated with early career attrition in this cohort.

The mechanisms underlying the observed association are likely multi-dimensional, involving physiological adaptation, psychological characteristics, and the socio-technical filters of talent identification. Early professional match exposure may reflect not only individual player readiness but also broader organizational factors. Players with substantial minutes before age 18 may benefit from accelerated tactical learning and professional socialization [11, 12], as well as greater access to elite club infrastructure, including structured strength and conditioning, medical supervision, and performance-monitoring systems [13, 14]. Consequently, early professional exposure may function partly as a proxy for institutional investment and support systems that promote professional continuity. From a developmental and environmental perspective, the survivor bias framework offers a compelling explanation for the observed results [15]. In high-performance systems, athletes are subject to intense selection pressures where only those possessing superior observed robustness and recovery capacity are selected for, and able to tolerate high volumes of exposure [15]. Consequently, the group with > 1,750 min likely represented a highly selected population that had already demonstrated an innate ability to withstand the physical rigors of senior competition. Rather than the exposure itself creating durability, high match volumes may serve as an indicator of a player’s existing robustness and their successful navigation of the youth-to-senior transition.

These observations are particularly relevant regarding the neuromuscular maturation occurring during late adolescence. Female athletes are biologically predisposed to higher rates of ligamentous injuries, most notably anterior cruciate ligament (ACL) ruptures, which peak in incidence during the mid-to-late teens [16]. While some athletes exposed to senior-level training may show more robust movement patterns [17], this study cannot disentangle whether this reflects a selection of individuals already possessing such patterns or the effects of structured training. This aligned with evidence suggesting that structured, high-volume training can be associated with reduced injury odds, provided that load increases are managed systematically [18, 19].

The influence of biological maturation status cannot be ignored when interpreting the success of early-debuting players. Early-maturing athletes frequently dominate youth selection processes due to temporary physical advantages [5]. In this study, the high-exposure groups debuted earlier and were significantly taller, suggesting that attraction advantages may influence early professional minute allocation [20]. However, the sensitivity analysis demonstrated that association between exposure and career sustainability remained significant even when adjusting for height. This suggests that while physical maturity may have been associated with the initial debut, long-term career sustainability is likely driven by other factors, such as the accumulation of technical and tactical expertise, and by pre-existing attributes that both enabled early selection and supported continued participation [21, 22].

Psychologically, the transition from youth to senior professional status represented a period of significant identity formation and stress [23]. Players who successfully navigated high-exposure roles before age 18 likely developed a robust athletic identity and high levels of perceived competence, which were critical buffers against sports burnout [23, 24].

Traditionally, the Early Sport Specialization (ESS) model has been associated with increased risks of overuse injury and premature retirement [25], leading to consensus recommendations to delay specialization [26]. Although we do not know whether the athletes in our cohort specialized early, these insights help contextualize developmental pathways and exposures in elite female soccer. The high attrition rate observed in the low-exposure group (48.5%) aligned with research into the youth-to-senior transition gap. Many youth players were retained by clubs primarily to facilitate the development of a small number of profit-maximizing prodigies, a phenomenon sometimes termed talent wastage [27, 28]. Players who debuted but failed to secure regular minutes (the ≤ 250-minute group) may have found themselves in a developmental limbo, leading to decreased motivation, a perceived lack of competence, and eventual dropout as they prioritized education or alternative careers [29].

Practically, these findings support the progressive integration of talented youth into professional play, as cumulative minutes above 750 before age 18 are linked to higher retention. Early professional minutes should be viewed as an indicator of readiness and load tolerance. Clinicians and coaches should use this information to guide load management while ensuring players receive the necessary medical and psychosocial support to sustain their development.

The primary strength of this study is its focus on professional female soccer players, a population comprising less than 20% of current soccer research [30]. By analyzing a large, multinational cohort across four major European leagues, this research provided high-powered evidence of a dose–response association between workload and longevity specific to women’s developmental trajectories.

The novelty of this study also stems from its granular quantification of professional minutes as a continuous exposure variable. Most prior research relied on categorical status (e.g., debuted vs. not debuted) or age of entry into academies [31]. By using thresholds (e.g., > 1,750 min), this study demonstrated a clear dose–response association that added significant detail to the understanding of workload and longevity. Furthermore, the use of sustained attrition (≥ 12-month absence) as a primary outcome provided a more definitive metric of career sustainability than contract status or participation, which are prone to seasonal fluctuations.

Finally, the longitudinal nature of the study and following players from their debut until age 23 captured the most volatile phase of the professional career. This period was characterized by simultaneous biological, psychological, and social transitions, including the shift from adolescent to adult competition and the potential move into higher education or full-time employment [5, 20–23].

Several methodological limitations should be acknowledged when interpreting these findings. First, this study relied on publicly available match-record databases that were not designed for epidemiologic research. The primary outcome was derived from a source that does not provide cause-specific information (e.g., injury, maternity leave, or retirement). Therefore, rather than being a clear indicator of career failure or any particular mechanism, the result should be interpreted as decreased professional continuity. Furthermore, a number of significant individual-level confounders, such as training load, maturation timing, socioeconomic factors, and past injury history, could not be adjusted. Second, the retrospective design and potential for incomplete capture are intrinsic to our data source and could introduce bias if missingness differs by exposure group. Finally, the study period coincided with rapid professionalization of women’s soccer, meaning that structural changes in medical support, financial security, and competitive standards may have influenced career sustainability differently across debut cohorts.

Despite these limitations, this study offers new evidence that increased exposure to early professional matches is an indication of longer career sustainability. Instead of limiting playing time in general, practitioners should focus on optimising transitional pathways through targeted injury prevention, organised load management, and strategic organisational support that balances long-term athlete development with competitive exposure.

## Data Availability

Data will be made available or reasonable request.

## Abbreviations

CI: Confidence Interval
RPE: Rating of Perceived Exertion
TRIMP: Training Impulse
SD: Standard Deviation
IQR: Inter-quartile Range
HR: Hazard Ratio
OR: Odds Ratio
ACL: Anterior Cruciate Ligament
ESS: Early Sport Specialization
